# Perioperative Antibiotic Prophylaxis in Pediatric Cardiac Surgery—Simple Is Better

**DOI:** 10.3390/antibiotics12010066

**Published:** 2022-12-30

**Authors:** Julia Burzyńska, Radoslaw Jaworski, Bohdan Maruszewski, Andrzej Kansy, Katarzyna Dzierżanowska-Fangrat

**Affiliations:** 1 Department of Clinical Microbiology and Immunology, Children’s Memorial Health Institute, 04-730 Warsaw, Poland; 2 Department of Pediatric Cardiothoracic Surgery, Children’s Memorial Health Institute, 04-730 Warsaw, Poland; 3 Department of Anesthesiology and Intensive Care, Faculty of Medicine, Medical University of Gdansk, 80-210 Gdansk, Poland

**Keywords:** perioperative antibiotic prophylaxis, surgical site infection, pediatric cardiac surgery

## Abstract

Pediatric cardiac surgery requires perioperative antibiotic prophylaxis (PAP) to reduce the risk of surgical site infections. However, the complexity of these procedures and the metabolic immaturity of children impede the establishment of PAP regimens that are both efficacious and in line with antimicrobial stewardship (AMS). In this study, we compared two PAP regimens: cefazolin with gentamicin (in a retrospective group) and cefazolin only (prospectively) in children undergoing elective cardiac surgery. In the prospective group, additional elements of AMS were introduced, i.e., restricted access to cefazolin and more diligent use of empirical antibiotics proceeded by consultation with an AMS team. The rate of surgical site infections (SSI), the scope of PAP deviations, and the postoperative use of antibiotics other than PAP within 30 days after surgery were analyzed. There were no significant differences in the rate of SSIs between the groups (3.9% vs. 1.2% in the prospective and retrospective groups, respectively (*p* = 0.35)). However, in the prospective group, the PAP violation was significantly reduced compared with the retrospective group (full compliance with the PAP regimen was 45.5% vs. 4.8%, *p* < 0.001, respectively). In addition, a reduction of postoperative antibiotic use was observed in the prospective group (0.991 vs. 1.932 defined daily doses, respectively).

## 1. Introduction

Every year, more than two thousand surgical operations for congenital heart defects are performed in Poland [1]. The results of a point prevalence study of healthcare-associated infections conducted in 2014 and 2015 suggested increasing rates of infections in patients undergoing cardiovascular surgery [2]. According to the same data, surgical site infections (SSI) were the second most common type of nosocomial infection [2]. Cardiac surgery procedures carry a particular risk of infectious complications due to additional factors related to extracorporeal circulation and hypothermia [3]. The use of perioperative antibiotic prophylaxis (PAP) significantly reduces the incidence of surgical site infections (SSI). However, in pediatric patients, PAP faces some challenges. Due to metabolic immaturity in children, it is difficult to determine the appropriate dosage of antimicrobial drugs and to predict their pharmacokinetics and pharmacodynamics [4]. As a result, there is no clearly defined effective regimen of perioperative prophylaxis in children undergoing cardiac surgery [5]. Concurrently, the growing antimicrobial resistance necessitates the prudent use of antibiotics in all fields, including PAP.

## 2. Results

A total of 84 and 77 patients in the retrospective and prospective groups, respectively, were included in the study. There were no differences regarding demographic features, i.e., age, sex, or body weight, nor in the total length of hospital stay between both groups. Colonization with methicillin susceptible *Staphylococcus aureus* was found in 12 (14.6%) and 7 (9.1%; *p* = 0.282) patients in the retrospective and prospective group, respectively, whereas colonization with methicillin resistant *Staphylococcus aureus* (MRSA) was not detected in any patient. In the prospective group, the mean duration of the operation, the duration of the extracorporeal circulation (ECC), and the duration of insertion of the aortic clamp were shorter than in the retrospective group (Table 1).

There were no significant differences in the concentrations of inflammatory parameters during the first three days after surgery between the groups (Table 2). Similarly, the frequency of surgical site infections did not differ between the groups: there were three (3.9%) SSIs and one (1.2%) SSI in the prospective and the retrospective group, respectively (*p* = 0.35).

An analysis of the most common deviations from the PAP regimen showed that, in the prospective group, the frequency of errors related to the administration of the first and second (if required) dose of cefazolin was significantly reduced (Table 3). In this group, 100% of the patients received their first dose of antibiotic, and nearly 80% of the patients received their first dose at the correct time and at the proper dosage. In the retrospective group, among the patients who received the first cefazolin dose, deviations in the dosage or timing of administration occurred in more than 50% of the cases. Additionally, in this group, the gentamicin dose was omitted in 28 (33.3%) patients. Importantly, PAP was prolonged to over 48 h in 23 (27.3%) patients in the retrospective group, but only in 9 (11.7%) patients in the prospective one (*p* = 0.009).

An important parameter in the postoperative assessment of patient management was the initiation of empiric antibiotics (antibiotics other than perioperative prophylaxis). In the prospective group, fewer patients received an empirical treatment within the first 5 days after the surgery, compared with the retrospective group (34; 27.3% vs. 23; 41%, respectively, *p* = 0.068), although the difference was not statistically significant. Additionally, the total quantity of antibiotic used within the 30 days after the surgery in the prospective group was over two times lower than in the retrospective group (0.991 vs. 1.932 defined daily doses, respectively).

## 3. Discussion

Since their discovery, antibiotics have played a key role in treating human infections. The antibiotic resistance of microbes is a natural phenomenon, but its acceleration by environmental pressure caused by the excessive and unreasonable use of antibiotics is one of the major problems of modern medicine and public health on a global scale [6,7]. Currently, the World Health Organization calls for action on antimicrobial stewardship worldwide [8]. Antibiotic surgical prophylaxis is one of these areas where there is still a lot of room for improvement.

The main goal of using antibiotics in perioperative prophylaxis is to reduce the incidence of surgical site infections (SSI), which are among the most frequent healthcare-associated infections [9,10]. To prevent SSI, a proper PAP protocol must be implemented and adhered to. However, some studies have shown that prophylactic regimens are violated, and that the prolongation of antibiotic administration is one of the most frequent deviations [2].

In this study, we compared two PAP regimens in pediatric patients undergoing elective cardiac surgery. The main difference between these schemes, introduced in the prospective group, was the elimination of one of the antibiotics administered immediately before surgery, i.e., gentamicin. Reducing the number and spectrum of activity of antibiotics given as a perioperative prophylaxis did not increase the incidence of surgical site infections or infections overall (as measured by the concentration of inflammatory markers within the first 3 days after the surgery or the need for empirical antibiotic treatment within the first 5 days after the operation) in the prospective group. However, the simplification of the PAP scheme resulted in a significant reduction in deviations from the protocol: significantly more patients received the required doses of cefazolin at the proper dosages and timing. Other pediatric studies also showed that simplification of the PAP guidelines was associated with better adherence to the protocol [11].

In addition, fewer patients in the prospective group had prophylaxis prolonged to over 48 h, which probably resulted from the restricted availability of cefazolin and extensive education before the implementation of the new PAP protocol. Studies in adults showed that extending the duration of perioperative prophylaxis did not translate into a reduction in the incidence of surgical site infections, but might lead to severe complications such as *Clostridioides difficile* infections or acute kidney failure [12,13]. Similarly, in cardiac surgery pediatric patients, shortening the duration of PAP to 48 h after the end of surgery or to 24 h after sternal closure did not increase the rate of SSI when compared with prolonged antibiotic administration until thoracic drain removal (5.8% vs. 6.2%, respectively) [14]. Some authors showed that, in neonates and infants, surgical prophylaxis in various (other than cardiac) procedures limited to 24 h had a beneficial effect on SSI rates when compared with 48 h PAP (9.1% vs. 16.9%, respectively) [15]. Other studies concerning pediatric cardiac surgery showed that PAP limited to 24 h did not significantly increase the rate of SSIs compared with prolonged (over 24 h) antibiotic administration (18.6% vs. 26.9%, respectively) [16].

The use of antibiotics in postoperative management was also analyzed, and a decreased frequency of initiation of empirical treatment was achieved in the prospective group, as well as a reduction of total antibiotic consumption in these patients. This most likely resulted from a more cautious use of these drugs, supported by an AMS member. Many studies revealed that AMS programs including easy access for consultations are efficacious in limiting unnecessary antibiotic use [17,18,19,20].

Our study has some important limitations. It was conducted in a single center with a relatively small number of patients included. Additionally, the PAP regimens were compared in a retrospective and prospective group, which differed slightly in terms of surgical features.

We cannot also exclude that. due to the shorter duration of operation and the use of ECC and an aortic clamp in the prospective group, the risk of postoperative infectious complications were different in both groups. This could influence the diagnostic and therapeutic approach regarding infection control and empirical antibiotic use. However, in our opinion, this hypothetical effect seems insignificant, since concentrations of inflammatory markers after the surgery did not differ between the groups.

In conclusion, our study showed that the simplification of the antibiotic prophylaxis regimen combined with a restriction of prophylactic drug availability did not increase the SSI rate, but was associated with better adherence to a PAP protocol. In addition, consultations with an AMS team regarding antibiotic use in the postoperative period led to diminished frequency of empiric treatment and a reduction of the total antibiotic consumption in cardiac surgery patients.

## 4. Materials and Methods

We performed a comparative study of two perioperative antibiotic prophylaxis regimens used in pediatric patients who underwent elective cardiac surgery. The study involved two groups: a retrospective group operated on in 2018, from 3 January to 4 June, in whom cefazolin in combination with gentamicin was used, and a prospective group operated in 2019, from 31 January to 14 June, in whom cefazolin was used solely. Patients with an allergy to penicillins were excluded from the study. The detailed PAP schemes are shown in Table 4. An additional change introduced in the prospective group involved a restricted use of cefazolin limited exclusively to the operating theatre and the surgical intensive care unit. Moreover, if an empiric antibiotic therapy after the surgery was to be initiated, physicians in the prospective group were encouraged to consult a specialist from the antimicrobial stewardship (AMS) team.

Data were initially collected for 200 consecutive patients (100 in each group) who received the standard PAP schemes in use in the respective period of the study. Subsequently, to ensure homogenous and comparable data, patients who underwent reoperation (11 in the retrospective group vs. 6 in the prospective group) or who had a delayed sternal closure (5 vs. 17, respectively) were excluded from the study.

In the prospective group, concentrations of serum C-reactive protein (CRP) and procalcitonin (PCT) were measured for three consecutive days after the surgery and compared with those in the retrospective group. The frequency of surgical site infections in both groups were analyzed according to the definitions of the “European Centre for Disease Prevention and Control: Point prevalence survey of healthcare-associated infections and antimicrobial use in European acute care hospitals-protocol version 5.3” (6). Additionally, in both groups, non-compliance to the PAP regimen was assessed (such as incorrect dosage and timing of the first and second cefazolin dose or an extension of the antibiotic perioperative prophylaxis to over 48 h). The consumption of antibiotics other than PAP within 30 days after an operation was compared between the groups.

### Statistical Methods

The normality of the data distribution was assessed using the Kolmogorov–Smirnov and Shapiro–Wilk W tests. Data that followed a normal distribution pattern and were analyzed using the t-test for equality of means. The equality of variances was estimated using Levene’s test. Data that did not follow a normal distribution were analyzed using the nonparametric Mann–Whitney U test. The relationships between categorical variables were assessed using the Pearson’s chi-squared test and, in the case of subgroups comprising less than 5 cases, a Yates’s correction was applied. Differences were considered significant when the *p*-values were below 0.05. For continuous variables, the mean value with standard deviation, as well as the range were evaluated. Categorical variables were described in terms of the number and percentage of each subgroup, and the respective values were rounded up to one decimal place. Statistical analysis was performed using SPSS version 20.0 (SPSS Inc., Chicago, IL, USA).

## Figures and Tables

**Table 1:** Demographic and surgical characteristics of children in both PAP regimens.

Feature	Retrospective Group	Prospective Group	*p* Value
Age (months) *	44.6 (58.9; 0.17–213)	49.3 (61.5; 0.2–218)	0.802
Gender, female	36 (42.8%)	41 (53.2%)	0.187
Body weight (kg) *	15.3 (16.7; 2.3–72)	16 (16.1; 1.8–72)	0.601
Total duration of hospital stay (days) *	35.8 (52.5; 8–438)	30.3 (36.9; 3–222)	0.311
MSSA nasal colonization	12 (14.6%)	7 (9.1%)	0.282
Intraoperative administration of steroids	53 (63.1%)	48 (62.3%)	0.921
Duration of the operation (minutes) *	259 (124; 65–615)	223 (102; 30–580)	0.049
Use of ECC	66 (78.6%)	65 (84.4%)	0.341
Duration of ECC (minutes) *	140 (58.5; 44–395)	112 (57; 35–342)	0.001
Duration of aortic clamp (minutes) *	64 (27; 11–135)	56 (31; 15–143)	0.035

* mean with standard deviation and range; kg—kilograms; MSSA—methicillin susceptible *Staphylococcus aureus*; ECC—extracorporeal circulation; SD—standard deviation.

**Table 2:** Inflammatory parameters values in the first three postoperative days.

Parameter	Postoperative Day	Retrospective Group; Mean (SD; Range)	Prospective Group; Mean (SD; Range)	*p* Value
Procalcitonin (PCT)	1	4.4 (10.2; 0.07–62)	6.4 (19.3; 0.07–140)	0.312
	2	6.8 (23; 0–162)	5.6 (15.7; 0.06–107)	0.924
	3	5 (16.3; 0–96)	7.1 (19.6; 0.08–112)	0.883
C-reactive protein (CRP)	1	4.4 (10.2; 0.65–91.8)	3.6 (2.4; 0.57–13.75)	0.495
	2	7.9 (10.8; 0.28–90.8)	6.7 (5.1; 0–25.6)	0.754
	3	7.5 (8.4; 0.83–59.32)	6.5 (5.8; 0–34.1)	0.643

SD—standard deviation.

**Table 3:** Assessment of preoperative and intraoperative compliance with the PAP regimen.

Feature	Retrospective Group	Prospective Group	*p* Value
Administration of the first dose of cefazolin	79 (94%)	77 (100%)	0.036
Correct dosage of the first cefazolin dose	38 (45.2%)	61 (79.2%)	<0.001
Time of administration of the first dose of cefazolin (minutes before the procedure) *	78.5 (74.5; 10–515)	40 (29; 0–170)	<0.001
Correct timing of the first dose of cefazolin	28 (35.4%)	60 (77.9%)	<0.001
Administration of the second dose of cefazolin (if required)	7 (15.6%)	17 (56.7%)	<0.001
Correct (appropriate) size of the second dose of cefazolin (if required)	4 (57.1%)	14 (82.4 %)	0.215
Time of administration of the second dose of cefazolin (minutes after the start of the procedure, if required) *	20 (112; 70–380)	201 (31; 155–260)	0.014
Correct timing of the second dose of cefazolin (if required)	3 (42.9%)	16 (94.1%)	0.014
Extension of the antibiotic perioperative prophylaxis to over 48 h	23 (27.3%)	9 (11.7%)	0.009
Full compliance with the PAP protocol	4 (4.8%)	35 (45.5%)	<0.001

* mean with standard deviation and range.

**Table 4:** Antibiotic prophylaxis schemes used in the study.

Retrospective Group	Prospective Group
Standard protocol	
Cefazolin: - dosing: 30 mg/kg iv - time of the first dose: 5–60 min before the procedure - additional cefazolin dose of 15 mg/kg added to CPB priming - redosing: every 4 h intraoperatively - postoperative dosing: 30 mg/kg iv every 8 h for max. 48 h (max. 6 doses) * and Gentamycin (single dose): - dosing: 4 mg/kg iv for newborns; 5 mg/kg iv for infants and older children - time of the first dose: within 60 min before the procedure	Cefazolin: - dosing: 30 mg/kg iv - time of the first dose: 5–60 min before the procedure - additional cefazolin dose of 15 mg/kg added to CPB priming - redosing: every 4 h intraoperatively - postoperative dosing: 30 mg/kg iv every 8 hours for max. 48 h (max. 6 doses) *
Alternative protocol and additional procedures	
Nasal colonization with MSSA or MRSA: - mupirocin 2% ointment 2–3 times/day for min. 3–5 days In patients with preoperative MRSA nasal colonization cefazolin was replaced by: - vancomycin 15 mg/kg iv (60 min infusion started 120 min before the procedure)	

* except for children with an open sternum, in the latter subgroup antibiotic prophylaxis up to 24 h after the closure of the sternum; CPB—cardiopulmonary bypass; MSSA—methicillin susceptible *Staphylococcus aureus*; MRSA—methicillin resistant *Staphylococcus aureus*.

## Data Availability

The data presented in this study are available on request from the corresponding author.
